# Shiga Toxin: Expression, Distribution, and Its Role in the Environment

**DOI:** 10.3390/toxins3060608

**Published:** 2011-06-14

**Authors:** Steven A. Mauro, Gerald B. Koudelka

**Affiliations:** 1 Department of Biological Sciences, Mercyhurst College, Erie, PA 16546, USA; Email: smauro@mercyhurst.edu; 2 Department of Biological Sciences, University at Buffalo, Buffalo, NY 14260, USA

**Keywords:** Shiga toxin, bacteriophage, *E.coli* O157

## Abstract

In this review, we highlight recent work that has increased our understanding of the production and distribution of Shiga toxin in the environment. Specifically, we review studies that offer an expanded view of environmental reservoirs for Shiga toxin producing microbes in terrestrial and aquatic ecosystems. We then relate the abundance of Shiga toxin in the environment to work that demonstrates that the genetic mechanisms underlying the production of *Shiga toxin* genes are modified and embellished beyond the classical microbial gene regulatory paradigms in a manner that apparently “fine tunes” the trigger to modulate the amount of toxin produced. Last, we highlight several recent studies examining microbe/protist interactions that postulate an answer to the outstanding question of why microbes might harbor and express *Shiga toxin* genes in the environment.

## 1. Shiga Toxin

Shiga toxin (Stx) is best known as an essential virulence factor in human disease mediated by *Escherichia coli* (*E. coli*) O157:H7. This strain was responsible for the first documented outbreak of haemorrhagic colitis in the US in 1982 [1]. Since then, the incidence of *E. coli* O157:H7-related disease has increased annually, with about 73,000 cases and 60 deaths currently occurring annually in the United States. The two antigenically distinct Stx variants (currently known as Stx1 and Stx2) produced by *E. coli* are related to the Shiga toxin produced by *Shigella dysenteriae* [2,3,4,5], which was first identified over 100 years ago [6]. The similarity is particularly high (one amino acid difference) between the sequences of *E. coli*-derived and the Stx of *S. dysenteriae*. In addition to *Shigella and E. coli*, *stx* variants have been shown to be expressed by *Enterobacter*, *Citrobacter*, *Acineobacter*, *Campylobacter*, and *Hamiltonella* bacterial species [7,8,9,10,11,12,13]. Thus, the genes encoding Shiga toxin may be broadly distributed among bacteria (see also below).

## 2. Shiga Toxin Encoding Bacteriophages

The genes for Stx in *S. dysenteriae* are located on the chromosome [14]. However, the Stx genes in *E. coli* are exclusively associated with active or cryptic lambdoid prophages. The enterohemorrhagic *E. coli* (EHEC) strain (*Escherichia coli* O157:H7 strain 933) that caused the first US outbreak of haemorrhagic colitis [1] encodes both Stx1 and Stx2. Genes encoding these toxins are located on different lambdoid bacteriophage that lyosgenize this strain. Bacteriophage that separately encode Stx1 and Stx2 have been isolated from various EHEC strains, and these phage are now representative of the Stx-encoding prophages. 

Since original isolation of Stx1 and Stx2 lambdoid bacteriophage from Shiga toxin encoding *E. coli* (STEC), many other Stx-encoding phages have been isolated and their sequences at least partially characterized. These data confirm that the Stx2-encoding phage have the general lambda-like organization of genes. The overall construction of Stx1 phages is similar to that of Stx2-encoding phages, and their genes display some similarity [15], whereas other Stx1 phages contain different sets of homologous genes.

Although homologous, the component genes of individual lambdoid phages are arranged modularly and are generally a mosaic construction of genes from a particular family of genes. This modular and mosaic structure extends to individual domains of single proteins. For example, the sequence of the DNA binding domain at the *N*-terminus of the repressor of phage 933W is nearly identical to that of the non-toxic lambdoid phage HK022, but the sequence of the *C*-terminal oligomerization domain of this protein is identical to that found in the repressor of the Stx1-encoding phage H19-B [16].

The opportunity to create new species via recombination is shown by analysis of the genomic sequence of the *E. coli* O157:H7 strain EDL933 genome [17]. This strain harbors two active bacteriophage, each expressing either *stx_1_* or *stx_2_* genes. This finding demonstrates that a STEC strain can harbor more than one Shiga toxin encoding bacteriophage. In addition, this strain is lysogenic for many cryptic phage genomes, providing ample material for recombination and a strategy for rampant spread of Stx in the environment. 

## 3. Shiga Toxin in the Environment

Microorganism diversity in the environment, coupled with the limits of protein detection in environmental samples, has made it difficult to directly and specifically detect Stx producing microorganisms outside the laboratory. Instead, amplification of bulk DNA isolated from a sample or specific culturing of *E. coli* or other microbial isolates have been used to test for the potential of a particular environmental sample to harbor microbes that express Stx. Despite the limitations in scope and specificity of these techniques, it is becoming apparent that Stx-producing microbes are plentiful and can exist in diverse environmental conditions.

## 4. Shiga Toxin in Terrestrial Environments

In terrestrial environments, cows have been thoroughly discussed as hosts for Stx producing bacteria that have been implicated in human illness through fecal contamination of beef, plants, and soils in the nearby environment [18,19,20,21]. The actual amount of Stx-producing microbes in cattle has been more difficult to ascertain. This is likely due, in part, to differences in techniques utilized for Stx detection. For example, using a Vero cell cytotoxicity assay, Stx was deemed to be absent in 100 fecal samples obtained from diarrheic calves [22]. In contrast, 89% of different cattle fecal samples tested positive for *stx* when phages were examined by qPCR [23]. A PCR approach of bacterially enriched cow fecal samples gives more consistent results across studies, with between 18%–25% appearing to be a general range of cow fecal samples that contain *stx* genes [24,25,26,27]. However, a similar approach for detecting *stx* genes in Brazilian dairy cattle feces was as high as 82% of the samples tested [28]. Moreover, in a study that analyzed the presence of *stx* genes in dairy cows and calves in Argentina, a detection frequency of *stx* in the cow feces sampled ranged from 4% to 60%, a variance that was dependent on the season in which the feces were collected [29]. Regardless of the variability of Stx detection in different studies, it is clear that cows are a major reservoir for Stx-producing microbes.

While cows have been considered the major carriers and transmitters of Stx-producing microbes on farms, it is becoming evident that other agriculturally based animals must now be considered in this regard. Sheep are one such example. In one study, over 100 fecal samples obtained from sheep and lambs were analyzed, and it was found that 87.6% of the samples tested positive for the *stx* gene [30]. Analysis of *E. coli* isolates obtained from sheep feces in another study found that 65.9% of the sheep tested positive for microbes carrying the *stx* gene [31]. Furthermore, *E. coli* O157 collected from sheep feces in a Netherland slaughterhouse were shown to both contain *stx* genes and to be cytotoxic to Vero cells, suggesting that these strains are able to produce Stx protein [32]. This research seems to indicate that sheep are similar to cows in their ability to harbor Stx-producing organisms. 

The presence of *stx* genes in farm animals extends beyond sheep and cows. For example, examination of bacterial strains obtained from the feces of goats demonstrated that nearly 75% of these animals contained microorganisms which have *stx* genes [33]. An analysis of *E. coli* strains isolated from goat feces provides a range between 10%–50% of goats that contain microorganisms which possess a *stx* gene, some of which have been shown to be toxic to Vero cells, implying that bacterial strains in some of these samples produce Stx protein [34,35,36]. Similar levels of *stx* genes and cytotoxic potential of STEC has been established in pigs and buffalos [37,38,39,40,41]. While there are only a few studies thoroughly examining the presence of *stx* in other farm animals such as chickens, turkeys, and rabbits, the microflora of the guts of these animals have also been implicated as reservoirs for Stx-producing microorganisms. [42,43,44,45]. Taken together, these recent studies provide strong evidence that farm animals other than cows have the potential to carry and spread Stx in the environment.

Recent studies have also solidified the notion that other animals, besides those involved in agriculture, harbor organisms with the potential to produce Stx. For example, over 50 STEC strains have been isolated from deer feces and shown to contain *stx* genes and exhibit Vero cell cytotoxicity [46,47]. In a survey of 50 fecal samples obtained from white tailed deer in Pennsylvania, it was found that nearly 50% of the samples contained a *stx* gene, suggesting that the presence of Stx-producing organisms in deer may be as common as they are in cows or other farm animals [48].

Evidence exists that implicates other animals as hosts for Stx-producing microorganisms, such as dogs, cats, wild boars, and various zoo animals; including yaks, alpacas, antelopes, and llamas [49,50,51]. Additionally, wild bird populations have been shown to harbor organisms which possess the *stx* gene. In a study that analyzed fecal samples from more than 2000 wild birds, 12 bird species contained feces that harbored microbes which tested positive for the *stx_1_* gene and 30 bird species contained bacteria in feces that tested positive for the *stx_2_* gene [52]. Additionally, airborne particulates in a contaminated building have been shown to contain bacteria with *stx* genes that have been linked to an outbreak of STEC in humans [53]. Taken together, these studies make it clear that Stx-producing organisms and highly abundant in a variety of terrestrial ecosystems. 

## 5. Shiga Toxin in Aquatic Environments

Aquatic environments have also been shown to harbor Stx-producing organisms that have been linked to a number of STEC outbreaks [54]. For example, Stx has been implicated as a possible pathogenic agent in drinking water responsible for gastrointestinal illness outbreaks. [55,56,57,58,59,60]. In all of these cases, the presence of either *stx_1_* and/or *stx_2_* genes was confirmed, but the actual amount of bacterial isolates that contained these genes in the water source was not identified. Surprisingly, very few studies exist that have attempted such an analysis in drinking water. One study that tested for the presence of *stx* genes in drinking water in India reported 30% of the 60 screened *E. coli* isolates from these water sources contained either *stx_1_* or *stx_2_* [61]. In a larger study conducted in Austria, over 200 *E. coli* isolates were obtained from various drinking water sources, and only one was found to contain *stx_2_* [62]. These limited studies set a wide range for which to compare *stx* distribution and abundance in other drinking water systems, information which is important given the prevalence of Stx-dependent illness arising from drinking water.

Water used for recreational purposes has also been found to contain organisms which produce Stx and cause human illness [63,64,65]. While more is known about the extent to which recreational water environments harbor organisms with the *stx* gene, the range in detection of this gene is just as diverse as it is for drinking water. For example, in Lake Erie tributary water samples in which bacterial DNA was isolated, only 0.78% of over 700 water samples tested positive for the *stx_2_* gene [66]. In a similar analysis in beach waters of Lake Erie, over 10% of the samples tested positive for the *stx_2_* gene [66]. When fecal coliform isolates obtained from water samples on the California coast line was probed for *stx* DNA, a similar frequency of detection was observed in comparison to the Lake Erie beach waters study [67]. In another study that examined PEG precipitated DNA from various water sources in Southern California, 18% of the 44 water samples tested positive for *stx* genes [68]. 

Some studies that have analyzed *stx* gene presence in bacterial isolates obtained from water samples indicate an even higher percentage of *stx* gene presence in recreational waters. For instance, *stx* genes were present in 22.7% of *E. coli* isolates obtained from the river Ganga, and greater than 50% of fecal coliform isolates tested positive for *stx_2_* DNA in river water in Maryland and river water samples obtained in Michigan and Indiana [69,70]. Using in situ PCR on membrane entrapped bacteria obtained from river water, it was found that as many as 10^5^ bacterial cells/mL of bacteria can carry the *stx_2_* gene [71]. Due to the different methodologies employed, it is difficult to compare *stx* gene or Stx producing organism abundance in these samples. However, it is apparent from these studies that *stx* gene distribution in aquatic ecosystems can be highly variable, likely owing to local conditions which impact organism survival, but also how these conditions might influence the expression of genes required for Stx production and proliferation of organisms which contain these genes (see bacteriophage developmental regulation section below for more detail). 

What might cause the sporadic and variable appearance of *stx* genes in drinking and recreational waters? One major factor appears to be the influx of non-point sources of bacterial pollution, such as wastewater [54]. Indeed, wastewater has been shown to be a major source of microorganisms that harbor and express *stx*. In municipal or urban sewage, it has been estimated that a *stx* gene is present in 1 out of every 1000 fecal coliforms [72,73]. One *stx* gene was detected in every 100 coliform colonies examined in animal wastewaters [72]. The frequency of *stx* positive *E. coli* has been shown to be even higher in non-sewage wastewaters. For example, in an analysis of *E. coli* isolates from 224 wastewater samples obtained from slaughterhouses in France, it was found that 13% tested positive for the *stx_2_* gene [74]. In agricultural waste lagoons, 25% of *E. coli* isolates tested positive for the *stx_2_* gene [75]. Given the high bacterial titers that can exist in these water samples, the total amount of organisms harboring and potentially capable of expressing *stx* is considerable. 

In addition to bacteria containing the *stx* gene in wastewater, there have also been several studies that document the presence of bacteriophages in wastewater which contain *stx* genes. For example, 70% of urban sewage samples and 94% of animal wastewater samples tested positive for phages that contain the *stx_2_* gene, which could reach values of up to 10^10^ gene copies/mL of wastewater [23,76]. The number of *E. coli* specific phages that contain a *stx* gene is lower, detectable in 16%–19% of phage plaques and estimated to occur at a frequency of 1–10 phages/mL of sewage water [77,78]. These studies demonstrate that both bacteria and bacteriophages which contain *stx* genes are abundant in wastewater. 

While wastewater has been attributed to pollution of recreation and drinking water in specific cases [60], the general efflux of these to a water supply remains unknown, especially in cases where water treatment facilities are in effect. Given the ability of *stx_2_-*encoding phages to persist under thermal stress and chlorination, the chronic release of these types of microbes to aquatic environments from wastewater remains a possibility [79]. However, attempts to relate *stx* gene or Stx-producing organism presence to indicators of wastewater or fecal pollution have failed to show a correlation in many cases [66,67,80]. This suggests that other factors besides general wastewater efflux explain the presence of microbes harboring or expressing *stx* in aquatic environments. 

Point source biotic and abiotic factors may be an alternative source of pollution of Stx-producing organisms in recreational waters. As an example, algae obtained from Lake Michigan was found to contain *stx* DNA in 25% of the samples analyzed [81]. While the total sample size in this study was small, the abundance of algae in the Great Lakes and other bodies of water, in addition to the high abundance and potential growth of *E. coli* that can occur on these algal mats, provides impetus for further studies to characterize the role algae has in bioaccumulation of Stx-producing microbes in aquatic environments [82]. *Stx* DNA has also been detected in sediment obtained from both seawater and freshwater [69]. In addition, enriched bacterial cultures obtained from various shellfish types from the coast of France tested positive for *stx* genes, suggesting that aquatic wildlife might also act as a point source reservoir for Stx-producing microorganisms in recreational waters [83]. 

Regardless of the reservoir, as with terrestrial ecosystems, there appears to be a great deal of variability in the degree to which water maintains Stx and the organisms that produce this protein. While local environmental factors certainly play a role in this, the ability of any environment to either maintain or increase Stx concentration will depend on the ultimate survival and proliferation of the organisms that produce Stx, which inevitably occurs at the level of gene regulation. 

## 6. Bacteriophage Development Regulates Shiga Toxin Production

Since the majority of environmental bacterial isolates that express Stx do so through expression of genes on lambdoid prophages, the spread of Stx in the environment is intimately linked to the genetic circuitry underlying these types of phage. All lambdoid bacteriophages share a common developmental program. Upon infection of a bacterial cell, the lambdoid phages choose between two developmental fates. The phage can grow lytically, thereby killing the host. Alternatively, in lysogenic growth, the phage chromosome is inserted into the host chromosome and is replicated along with it, until a signal that induces lytic growth is perceived by the lysogenized cell. The genes for Stx are encoded in the late region of the phage. Late genes are only expressed while the bacteriophage is growing lytically. Therefore, Stx is not produced when the bacteriophage is in its lysogenic state. Hence, the *stx* genes reside harmlessly within a lysogenic phage in the bacterial host until induction of the phage causes Stx to be produced. 

In the known *stx*-encoding phages, the *stx* genes are located downstream from P_R_’. This promoter is only active during lytic growth and thus suggests an explanation as to how the lysis-lysogeny decision controls Stx production [84,85,86]. When the phage lytic program is induced, transcription initiates at the early promoters P_L_ and P_R_. Subsequent to transcription of the N gene from P_L_ and its translation, N activity permits an RNA polymerase transcribing from P_R_ to read-through transcription terminators and extend transcription into the *Q* gene. *Q* binds DNA at a site partly overlapping P_R_’ and acts as an anti-terminator thereby allow RNA polymerase to transcribe an operon that includes the *stx* genes [84,85,86,87,88]. 

As a result of its downstream position in the lytic cascade, synthesis of *Q* (and thereby Stx) depends on the activities of the early lytic promoters P_R_ and P_L_. In a lysogen these promoters are repressed by repressor DNA binding. During lysogen induction, repressor is inactivated, leading to expression of *Q*. Thus, synthesis of Stx is ultimately controlled by factors that influence repressor activity. Stx is not exported through any bacterial secretory machinery. Therefore its release from the cell depends on phage-encoded functions that cause bacterial lysis. Since *Q* also allows read-through of terminators that inhibit transcription of phage genes that catalyze host lysis, Stx release is also regulated by repressor activity. 

In addition to bacteriophage functions, early studies identified a promoter consensus sequence (*p*Stx) upstream of the *stxA* gene in Stx1-encoding bacteriophages. As a consequence of regulation by the iron sensitive repressor Fur, Stx1 production is repressed by iron [89]. The Fur operator site is not found in promoters encoding phage associated Stx2, hence iron availability does not regulate Stx2 production [90]. 

## 7. Repressor Regulation of Shiga Toxin Expression

As outlined above, the phage’s decision between lytic and lysogenic growth and therefore ultimate production of Stx is regulated by the gene regulatory activities of the phage-encoded cI repressor protein. The “escape” of the prophage from the host requires the inactivation of the repressor protein. To permanently inactivate this protein, the phage takes advantage of part of the host’s SOS response, the pathway involved in general response to DNA damage. In particular, the interaction of the repressor with RecA, the master regulator of the SOS response, stimulates the intrinsic autoproteolytic activity of the phage’s repressor protein. RecA-stimulated self-cleavage separates repressor’s *C*-terminal oligomerization domain from its *N*-terminal DNA binding domain [91,92,93,94,95] eliminating its ability to bind DNA, de-repressing the genes needed for lytic growth, which ultimately enables production of Stx.

Two observations demonstrate that the regulation of repressor levels *via* the SOS pathway plays an important role in regulating the production Shiga toxin in mammals infected with Shiga toxin encoding *E. coli* (and presumably other Stx encoding bacteria in the environment). First, reactive oxygen species like H_2_O_2 _ or superoxide, generated and released by leukocytes and neutrophils activates the SOS response in Stx encoding *E. coli* (STEC) leading to toxin release [96] and subsequent death of the “attacking” eukaryotic cell. A similar mechanism apparently allows Stx to be released from STEC in response to predation by single-celled eukaryotic predators [97], (see also below). Second, SOS-inducing antimicrobial agents (e.g., quinolones, trimethoprim) induce Stx gene expression [98]. At concentrations above those required to inhibit bacterial replication the SOS-inducing antimicrobial agents induce transcription of *stx* gene by up to 140-fold. Other antimicrobial compounds caused smaller, but nonetheless significant derepression of *stx* expression.

Aside from SOS induction by stimulated external agents, prophages in culture produce free phage in the absence of an inducing agent, in a process known as spontaneous induction. The fraction of lysogens that induce spontaneously is low compared to the number induced when the same number of lysogens is treated with an inducing agent. Nonetheless, this spontaneous induction depends on the presence of active RecA and hence is also mediated by changes in repressor levels. Analysis indicates that the subpopulation of STEC lysogens that spontaneously induce plays a major role in the production and release of Stx [99,100].

The frequency of DNA damage-independent (spontaneous) induction is much higher in *stx*-encoding bacteriophages than in other related bacteriophages [101]. The increased induction frequency in these *stx-*encoding phages has been attributed to the requirement for lower concentration of active RecA necessary for induction [101]. The critical level of RecA needed for induction of these phages could be determined by an increased sensitivity of the repressor to RecA, a decrease in the strength of repressor binding interactions with its operators, and/or a decreased total amount of repressor present in the lysogen. 

Recent work showed that the absolute affinities of 933W repressor for its DNA sites are not dramatically different than the affinities of other lambdoid phage repressors for their cognate operators. Analysis of transcription in bacteriophage 933W indicates that the amount of repressor is lower in 933W lysogens than in bacteriophage lysogens that do not normally encode Stx. Therefore the increased sensitivity of spontaneous induction frequency of Stx-encoding lysogenic phage is apparently due, at least in part, to a lower amount of repressor in the 933W lysogen than in λ lysogens.

Repressor levels in a lambdoid phage lysogen are regulated by autogenous positive and negative control. That is, repressor binding to one subset of binding sites leads to activation of repressor synthesis, whereas repressor occupancy of additional sites leads to repression of repressor expression. In the well studied non-STEC lambdoid phages, differential site binding by repressor and hence repressor gene activity depends on the cooperative binding of the repressor to multiple sites, some of which are separated by up to 2.5 kb. However, the repressor of *stx2*-encoding bacteriophage 933W does not bind DNA cooperatively [102]. This is apparently due to the highly divergent sequence of this repressor’s *C*-terminal oligomerization domain, a domain it shares with the repressor of the *stx1*-encoding bacteriophage H-19B. Therefore, bacteriophage 933W (and presumably bacteriophage H-19B as well) has evolved an alternative way to autogenously regulate repressor levels in a lysogen. The key feature of this alternative mechanism is the large difference in the relative affinities of 933W repressor for its DNA binding sites compared with that displayed by other phage repressors [102]. As a consequence in bacteriophage 933W, there is an extremely narrow range of concentration between the amounts of repressor needed to activate and repress 933W repressor synthesis.

## 8. Role of Bacteriophage-Encoded Shiga Toxin in the Environment

Surveys of bacterial genome sequences reveal that the genomes of both cryptic and active temperate phage are found with surprisingly high frequency in the chromosomes of these organisms. Although these toxins do affect humans and other mammals, these phage-encoded exotoxin genes are found at high frequencies in free phages and lysogenic bacteria isolated from environments where the presumed corresponding targets are not prevalent [103]. The prevalence of integrated bacteriophage DNA is surprising because the ultimate goal of bacteriophages is to reproduce, and in doing so, these phages kill their host. Consequently, the prevalence of phage DNA, especially phages encoding exotoxins, inside host chromosomes suggests that these toxin-carrying phages are tolerated because their presence provides an evolutionary advantage to the host. If the phage-borne exotoxins like Stx provide a bacterial population with the ability to combat predation, this would provide an explanation for the prevalence of phage-encoded exotoxin genes in the biosphere. These observations have led to the hypothesis that humans and other susceptible mammals are neither the original nor primary “targets” of these toxins [104].

Consistent with this idea, the genes encoding exotoxins are rarely integrated into the host chromosome, but instead are virtually always located on active bacteriophage. The exotoxins kill eukaryotic cells by means of receptors and pathways that are generally conserved among eukaryotic organisms. Utilization of a phage-encoded exotoxin as an anti-predator defense is cost-effective for the bacterial population since sacrificing a few cells to produce a toxin to kill a population’s major predators ultimately ensures the survival of the greater bacterial population. Inherent in this idea is that the anti-predator strategy may involve an “induction factor” to signal the presence of the predator. Therefore, the toxin gene can be carried by the host bacteria in a harmless, non-toxic, lysogenic state until its production is stimulated (induced) by the presence of a predator.

This hypothesis was recently tested by exploring how the presence of an exotoxin-encoding bacteriophage resident within bacteria influences the growth and survival of the bacterial population and a model unicellular eukaryotic predator, *Tetrahymena thermophila* [97]. When co-cultured with *Tetrahymena*, Stx-encoding bacteria kills this predator. Stx-encoding strains are less efficiently predated than are strains that do not encode this exotoxin. 

Bacteria appear to sense the presence of the *T. thermophila* by detecting the presence of reactive oxygen species (ROS) released by this organism via activation of the bacterial SOS system and consequently respond by inducing the synthesis of Stx by RecA-mediated cleavage of the phage repressor. Inactivation of this repressor also leads to phage lytic growth and subsequent host cell lysis, allowing Stx to be released into the environment. Indeed, the need to more readily respond to a predatory attack may be the evolutionary force that drives Stx-encoding bacteriophage to behave as “hair-triggers”, leading the alteration in the mechanism by which lysogeny is maintained in these phage. The ROS-mediated induction of phage growth, Stx synthesis, and release and subsequent Stx-mediated death of the ROS-releasing cells foreshadows the situation found in the mammalian response to bacterial infection. In this case, ROS generated and released by leukocytes and neutrophils activates the SOS response in Shiga toxin encoding *E. coli*, leading to toxin release [96] and subsequent death of the “attacking” eukaryotic cell.

In using the phage-encoded Stx as an antipredator defense, the lysogenic bacterial host dies. Therefore, fitness benefits of this antipredator defense mechanism do not accrue to the individual organism but to the overall bacterial population-a population that would include cells that are not lysogenic for the toxin-encoding bacteriophage. At first glance this strategy may seem overly “altruistic”. However, the genes encoding this exotoxin are found on mobile, temperate bacteriophage. Stx-encoding phages lysogenize naïve hosts at low frequency [101]. Phage-mediated killing of the naïve hosts would reduce the benefit of Shiga toxin’s antipredator activities on the fitness of this segment of the bacterial population. Lytic growth in the naive hosts does increase the amount of Stx produced [105], thereby amplifying the predator killing capacity of the original sacrificed cell. Hence, the presence of toxins genes, in this case Stx, on the temperate phages and linkage of toxin expression to lytic growth may provide substantial advantages to bacterial lineages that “choose” to harbor these phage. Utilization of an exotoxin encoded on an inducible phage is then a cost-effective defensive strategy for the bacterial population. These lysogenic populations sacrifice a few cells to produce a toxin that kills its major predator and produce infectious phage that have the potential to eliminate bacterial competitors [106]. This realization may help explain the apparent ubiquity and growing prevalence of Stx-encoding bacteria and phages within the biosphere.

It is unknown how many of the microbial predators are sensitive to Stx-mediated killing. However, it is clear that since predation by bacterivorous protozoa can substantially alter the composition of bacterial populations [107,108,109,110], the presence of cytolethal Stx releasing bacteria can be imagined to have substantial impacts on not just the types and numbers of bacteria present within microbial populations, but also the numbers and types of single-celled eukaryotes present as well. Future studies examining the environmental factors that activate the genetic machinery to produce Stx, and the consequence this has on other microbes within an ecosystem, are needed to provide greater clarity regarding the functionally of Stx in the environment. Only then can we gain a firmer understanding as to why Shiga toxins are so highly variable, yet abundant, in the biosphere. 
